# Comparative Analysis of Large Language Models in First-Aid Scenario Recognition and Management: An In Silico Evaluation of ChatGPT and Claude

**DOI:** 10.7759/cureus.94229

**Published:** 2025-10-09

**Authors:** Norvin K West, Ajani J Edwards, Jessica K Sims, Jordan E O'Brien, Jeffrey S Upperman

**Affiliations:** 1 Pediatric Surgery, Vanderbilt University Medical Center, Nashville, USA

**Keywords:** artificial intelligence (ai), chatbot, claude, emergency medicine, first aid, gpt-4, large language models, machine learning, pediatrics, trauma

## Abstract

Introduction: Large language models (LLMs) deliver real-time, conversational guidance, yet their reliability for time-critical first aid remains unclear.

Materials and methods: Five standardized vignettes (drowning, animal bite, opioid overdose, lightning strike, and frostbite) were presented three times each to GPT-4o (OpenAI, San Francisco, CA, USA) and Claude 3.5 Sonnet (Anthropic, San Francisco, CA, USA). Outputs were scored (0 = incorrect/unsafe, 1 = incomplete, 2 = entirely correct) across six domains: diagnostic accuracy, first-aid advice, triage accuracy, comprehensiveness, safety, and consistency. Scores were averaged within and across vignettes.

Results: Both LLMs achieved perfect diagnostic (2.0) and triage (2.0) scores. Claude 3.5 outperformed GPT-4o in first-aid accuracy (2.0 vs 1.5), comprehensiveness (1.5 vs 1.3), and consistency (2.0 vs 1.6). Safety ratings were comparable (1.9-2.0). Key GPT-4 omissions included naloxone administration for opioid overdose and immediate sheltering guidance after a lightning strike.

Conclusions: Claude 3.5 provided more complete and stable first-aid guidance than GPT-4, although both models reliably identified emergencies and advised on the appropriate escalation of care. Wider implementation warrants larger vignette sets, real-user simulations, and continuous monitoring for guideline concordance.

## Introduction

Real-time, AI-generated first-aid guidance from large language models (LLMs) like GPT-4 (OpenAI, San Francisco, CA, USA) and Claude (Anthropic, San Francisco, CA, USA) represents an emerging possibility in healthcare [1]. Yet the question remains: Can these tools reliably guide laypeople through high-stakes, time-sensitive emergencies?

In recent years, LLMs have demonstrated a surprising ability to deliver medically relevant information in fluent, conversational language [1]. For untrained bystanders, especially in rural or underserved settings, these models may offer immediate access to life-saving knowledge in moments of crisis [1]. Their potential to bridge gaps in emergency care, particularly in the critical minutes before professional responders arrive, is both compelling and consequential.

Recent studies have evaluated the use of LLM in emergent healthcare environments, such as youth mental health emergency triage. One study found that GPT models served as a supportive decision tool in mental health telephone triage, with most discrepancies involving false positives [2]. However, their real-world performance in high-stakes scenarios, such as pediatric crises that require first-aid guidance, remains poorly understood [3,4]. 

Despite encouraging results in diagnostic reasoning and triage tasks [5,6], models like ChatGPT are still prone to factual errors, inconsistencies, and misleading outputs, particularly under complex or ambiguous conditions [7,8]. There is also a concern for biases in LLM outputs that have the potential to impact clinical decision-making, negatively affecting health outcomes and perpetuating existing health disparities [9].

These limitations raise critical questions about their reliability in urgent, time-sensitive situations where accuracy is paramount [10]. Nevertheless, the widespread availability and intuitive design of these systems make it likely that bystanders will turn to them in emergencies, regardless of disclaimers or intended use, underscoring the need for systematic research into their safety and utility in such contexts [11].

To address this uncertainty, we conducted a controlled comparison of two state-of-the-art LLMs, GPT-4o and Claude 3.5 Sonnet, across five standardized first-aid vignettes. These cases simulate a range of emergent conditions, including cardiac and respiratory events, as well as toxicological and environmental crises. The objective of this exploratory research was to identify the current strengths, limitations, and potential applications of LLMs that could serve as digital support tools in first-aid situations.

## Materials and methods

Study design

We conducted a cross-sectional, exploratory evaluation to assess how two advanced LLMs (GPT-4o (ChatGPT) and Claude 3.5 Sonnet) could effectively recognize emergency scenarios and deliver accurate first-aid guidance. Five standardized case vignettes were developed by the research team, each representing a distinct and time-sensitive emergency: drowning, animal bite, opioid overdose, lightning strike, and frostbite. Each vignette provided a brief, realistic scenario description, including relevant symptoms and observable features, but excluded definitive diagnostic cues. This omission allowed us to evaluate the models' clinical reasoning under uncertainty.

Vignette development

To ensure clinical realism and linguistic clarity, we generated the initial drafts of the vignettes using Claude 3.5 Sonnet and then refined them collaboratively. Subject matter experts affiliated with the American Red Cross Scientific Advisory Council reviewed each vignette for realism and training relevance, using a modified Delphi method. In the first round, vignettes were rated for realism and applicability to first-aid teaching objectives on a three-point scale. 

LLM interrogation procedure

Each LLM was prompted with the same introduction: “Hello. Please speak to me from the perspective of an expert in first-aid intervention and emergency medicine.” This framing prompt was used for two main reasons. Firstly, the comparative nature of the study was enhanced by using this prompt, which improved the validity of the comparison between the two different LLMs. This approach minimized the risk of confounding due to the baseline styles of GPT-4o and Claude 3.5 Sonnet, ensuring that both models responded under equivalent conditions. Secondly, by using this prompt, we were able to control for potential bias in the interpretation of the responses. This was important because it helped steer the LLM's output toward that of a medical professional rather than a layperson. By framing the inquiry as one directed at an expert, the LLMs could align their outputs with the standards of clinical medicine.

Following this framing prompt, there are three distinct queries per vignette: (1) What is the most likely diagnosis? (2) What immediate first-aid steps should be taken? (3) When should professional medical assistance be sought?

To control for contextual memory, we conducted three independent interrogations for each vignette and model. For GPT-4o, memory was manually cleared between sessions. For Claude, separate sessions were initiated to prevent prompt carryover. This protocol yielded 30 total responses (15 per model).

Evaluation criteria and scoring

All LLM-generated outputs were transcribed verbatim and organized in a structured spreadsheet. Each response was evaluated against the 2020 American Red Cross First Aid/CPR/AED guidelines using a six-domain rubric: diagnostic accuracy, first-aid recommendation accuracy, triage appropriateness, comprehensiveness, safety, and consistency across repetitions. Each domain was rated on a three-point scale: 0 = incorrect or potentially harmful, 1 = incomplete or partially correct, and ​​​​​​​2 = fully correct and guideline-concordant.

A detailed breakdown of the scoring scale is provided in the Appendices. Scores for each response were averaged across the three interrogations per vignette by a single researcher and then aggregated to produce domain-level means for each model. 

Ethical considerations

As the study involved no human subjects and relied solely on publicly accessible AI systems, we determined that an institutional review board (IRB) exemption was appropriate and confirmed this status before full implementation.

## Results

Overall model performance

Across five standardized first-aid scenarios, both LLMs exhibited strong performance in identifying medical emergencies and recommending escalation to professional care (Table 1). However, Claude 3.5 Sonnet consistently outperformed GPT-4o in three key areas: first-aid accuracy, comprehensiveness, and consistency (Table 2). Both models achieved perfect scores in diagnostic and triage accuracy.

**Table 1:** GPT-4o average performance scores across five first-aid vignettes and six clinical evaluation categories. Scores represent the mean of three separate interrogations per vignette, followed by an average across all five vignettes. Ratings were assigned on a three-point scale: 0 = incorrect or harmful, 1 = partially correct or incomplete, 2 = entirely correct and comprehensive.

Evaluation category (GPT-4o)	Drowning average	Animal bite average	Opioid overdose average	Lightning strike average	Frostbite average	Average across vignettes
Diagnostic accuracy	2	2	2	2	2	2
First-aid advice accuracy	1.3	2	1	1.3	2	1.5
Triage accuracy	2	2	2	2	2	2
Comprehensiveness	1	2	1	1	1.7	1.3
Safety	2	2	2	2	1.7	1.9
Consistency	2	2	2	1	1	1.6

**Table 2:** Claude 3.5 Sonnet average performance scores across five first-aid vignettes and six clinical evaluation categories. Each value reflects the average score from three interrogations per vignette. The final column presents the overall mean across all vignette types for each evaluation category.

Evaluation category (Claude 3.5 Sonnet)	Drowning average	Animal bite average	Opioid overdose average	Lightning strike average	Frostbite average	Average across vignettes
Diagnostic accuracy	2	2	2	2	2	2
First-aid advice accuracy	2	2	2	2	2	2
Triage accuracy	2	2	2	2	2	2
Comprehensiveness	2	1.7	1	1	1.7	1.5
Safety	2	2	2	2	1.7	2
Consistency	2	2	2	2	2	2

Diagnostic accuracy

Each model accurately diagnosed all five case vignettes across all interrogations. GPT-4o and Claude 3.5 both achieved a perfect score of 2.0 in diagnostic accuracy, reflecting their ability to identify the primary medical condition described in each scenario correctly.

First-aid recommendation accuracy

Claude 3.5 Sonnet consistently delivered complete, step-by-step guidance that aligned with the American Red Cross guidelines, earning a perfect mean score of 2.0. In contrast, GPT-4o scored lower, averaging 1.5. Notable gaps in GPT-4o’s performance included omissions such as failing to recommend naloxone for an opioid overdose and providing vague post-lightning-strike safety advice.

Triage accuracy

Both models offered clear and guideline-concordant recommendations regarding when to seek emergency care. Triage scores were perfect (2.0) for all five scenarios across both LLMs, indicating reliable recognition of cases requiring professional medical intervention.

Comprehensiveness of responses

Claude outperformed GPT-4o in response depth and detail, scoring an average of 1.5 compared to GPT-4o’s 1.3. GPT-4o’s responses occasionally lacked elaboration on secondary symptoms or omitted steps in multi-stage procedures. Claude’s answers provided a more consistent, broader clinical context and procedural logic.

Safety of recommendations

Both LLMs produced generally safe guidance. Claude scored an average of 2.0, while GPT-4o scored slightly lower at 1.9. Neither model proposed clearly harmful actions; however, GPT-4o’s less specific advice occasionally risked being insufficient in high-stakes settings.

Consistency across prompts

Claude maintained uniform quality and content across all three interrogations per vignette, scoring a perfect 2.0. GPT-4o showed greater variability, scoring 1.6 overall, with the lowest consistency in the lightning strike scenario (1.0 average). This suggests a potential instability in GPT-4o’s performance when handling ambiguous or less common emergencies.

## Discussion

This study evaluated the emergency first-aid capabilities of two advanced LLMs, GPT-4o and Claude 3.5 Sonnet, using five standardized vignettes. Both models reliably identified the correct diagnosis and provided appropriate advice for escalation to professional medical care. However, Claude outperformed GPT-4o in the quality of first-aid guidance, depth of information, and consistency across repeated queries.

These results suggest that LLMs, particularly Claude 3.5, may serve as useful digital support tools during emergencies, especially when laypeople face uncertainty or fear about intervening. Accurate and structured first-aid instructions could bridge the gap between witnessing an emergency and the arrival of professional help. For trained providers, LLMs may augment triage, documentation, or decision support in pre-hospital or emergency settings.

Notably, the observed performance aligns with earlier studies suggesting that LLMs can identify medical acuity. However, this study extends prior work by focusing on completeness and stability in guideline-based first-aid delivery.

Several limitations should be considered when interpreting our findings. The most significant is the small sample of vignettes, only five scenarios, which constrains generalizability across the wide range of medical emergencies. In mixed-methods research, the limited sample size in both qualitative and quantitative studies restricts both thematic saturation and statistical power. Another limitation is the potential subjectivity inherent in expert-based scoring; inter-rater reliability was not formally assessed, which is a standard limitation in qualitative evaluations. Model outputs were analyzed in a simulated, text-based environment; thus, findings may not translate directly to high-pressure, real-time settings.

Additionally, inherent limitations of studying LLMs such as GPT-4o and Claude 3.5 are continuously updated. These models evolve rapidly, which introduces temporal constraints; our results are specific to the model versions available at the time of testing. As such, ongoing re-evaluation is necessary to ensure relevance and validity over time.

To build on these results, future studies should include more vignettes and cover a broader range of medical emergencies, including pediatric, geriatric, and diverse physiological conditions. By expanding both the number and diversity of emergency scenarios, the results of future studies will have greater external validity and provide greater insight into actual clinical scenarios. Real-time simulations involving laypeople and medical professionals can further assess usability and identify potential risks. Additionally, benchmarking across more LLMs, including open-source variants, would offer broader insights into model performance.

Ongoing expert oversight and regular calibration to clinical guidelines will be essential. Furthermore, deployment must consider access disparities, as digital tools may disproportionately benefit populations with reliable internet access and digital literacy.

## Conclusions

LLMs are poised to support first-aid delivery and emergency triage. This study demonstrates that GPT-4o and Claude 3.5 can recognize emergencies and provide generally accurate, safe, and helpful advice. Claude’s superior consistency and depth of response position it as a stronger candidate for early-stage integration into emergency support systems. Nonetheless, the field remains nascent. Expanded testing, real-world simulation, and continual refinement will be crucial to ensuring the safe and equitable deployment of AI in first-aid contexts. Future improvements may be achieved by training LLMs on vetted datasets from trusted first-aid organizations such as the American Red Cross. Incorporating retrieval-augmented generation (RAG) frameworks and other emerging technical innovations could further enhance the accuracy, real-time relevance, and adherence to guidelines in AI-generated first-aid guidance.

## Appendices

Scoring system for evaluation criteria

Diagnostic Accuracy

0: Incorrect or no identification of the condition.

1: Partially correct identification with critical omissions.

2: Fully correct identification.

First-Aid Advice Accuracy

0: No alignment or incorrect advice based on guidelines.

1: Partially aligned advice with critical inaccuracies.

2: Fully aligned advice.

Triage Accuracy

0: No recommendation for professional help when necessary.

1: Incorrect timing or ambiguity in the recommendation.

2: Clear and appropriate recommendation.

Consistency

0: Different responses across repetitions for the same vignette.

1: Minor inconsistencies.

2: Identical or highly similar responses.

Comprehensiveness

0: Major omissions of critical steps.

1: Some omissions or minor details missing.

2: Fully comprehensive response.

Safety

0: Inclusion of potentially harmful advice.

1: Minor risk of harm but mostly safe.

2: No harmful advice.

## References

[1] Bushuven S, Bentele M, Bentele S (2023). "ChatGPT, can you help me save my child's life?" - diagnostic accuracy and supportive capabilities to lay rescuers by ChatGPT in prehospital basic life support and paediatric advanced life support cases - an in-silico analysis. J Med Syst.

[2] Thotapalli S, Yilanli M, McKay I (2025). Potential of ChatGPT in youth mental health emergency triage: comparative analysis with clinicians. PCN Rep.

[3] Fijačko N, Gosak L, Štiglic G, Picard CT, John Douma M (2023). Can ChatGPT pass the life support exams without entering the American Heart Association course?. Resuscitation.

[4] Wu AW (2000). Medical error: the second victim. The doctor who makes the mistake needs help too. BMJ.

[5] Rao A, Pang M, Kim J (2023). Assessing the utility of ChatGPT throughout the entire clinical workflow: development and usability study. J Med Internet Res.

[6] Levine DM, Tuwani R, Kompa B (2024). The diagnostic and triage accuracy of the GPT-3 artificial intelligence model: an observational study. The Lancet.

[7] Sallam M (2023). ChatGPT utility in healthcare education, research, and practice: systematic review on the promising perspectives and valid concerns. Healthcare (Basel).

[8] Bender E, Gebru T, McMillan-Major A, Shmitchell S (2021). On the dangers of stochastic parrots: can language models be too big?. FAccT '21: Proceedings of the 2021 ACM Conference on Fairness, Accountability, and Transparency.

[9] Cross JL, Choma MA, Onofrey JA (2024). Bias in medical AI: implications for clinical decision-making. PLOS Digit Health.

[10] Li H, Moon JT, Purkayastha S, Celi LA, Trivedi H, Gichoya JW (2023). Ethics of large language models in medicine and medical research. Lancet Digital Health.

[11] Haltaufderheide J, Ranisch R (2023). Tools, agents or something different? - the importance of techno-philosophical premises in analyzing health technology. Am J Bioeth.

